# Sensitive Detection of Various Forms of Hydrogen Sulfide via Highly Selective Naphthalimide-Based Fluorescent Probe

**DOI:** 10.3390/molecules28176299

**Published:** 2023-08-28

**Authors:** Daniel Słowiński, Małgorzata Świerczyńska, Jarosław Romański, Radosław Podsiadły

**Affiliations:** 1 Institute of Polymer and Dye Technology, Faculty of Chemistry, Lodz University of Technology, Stefanowskiego 16, 90-537 Lodz, Poland; daniel.slowinski@dokt.p.lodz.pl (D.S.); malgorzata.swierczynska@dokt.p.lodz.pl (M.Ś.); 2 Department of Organic and Applied Chemistry, Faculty of Chemistry, University of Lodz, Tamka 12, 91-403 Lodz, Poland; jaroslaw.romanski@chemia.uni.lodz.pl

**Keywords:** hydrogen sulfide, fluorescent detection, naphthalimide-based probe, organic H_2_S donors

## Abstract

Hydrogen sulfide (H_2_S) is an important gasotransmitter, but only a few methods are available for real-time detection. Fluorescent probes are attractive tools for biological applications because of their high sensitivity, convenience, rapid implementation, noninvasive monitoring capability, and simplicity in fluorescent imaging of living cells and tissues. Herein, we report on a pro-fluorescent probe, NAP-Py-N_3_ based on naphthalimide derivative, which was found to show high selectivity toward H_2_S over various other analytes, including biothiols, making it feasible to detect H_2_S. After reaction with H_2_S, this probe showed rapid and significant turn-on green fluorescent enhancement at 553 nm (about 54-fold, k_2_ = 9.62 M^−1^s^−1^), high sensitivity (LOD: 15.5 nM), significant Stokes shift (118 nm), and it was found that the fluorescence quantum yield of fluorescence product can reach 0.36. Moreover, the probe has also been successfully applied to detect the gaseous H_2_S and to confirm the presence of H_2_S released from modern organic donors, which in recent years have been commonly used to investigate the role of H_2_S in biological systems. All the results indicate that this probe is excellent and highly valuable.

## 1. Introduction

The volatile organosulfur species are well identified for their characteristic obnoxious odor and are considered as toxic to human health and the environment [1]. Hydrogen sulfide (H_2_S) is the third reported endogenous gasotransmitter, along with CO and NO, and is regarded as one of the essential molecules of reactive sulfur species (RSS) [2,3]. Although it is a toxic, highly flammable, explosive, and notorious air pollutant gas [4], in the cells of living organisms it plays a relevant role in numerous pathological and physiological processes, including cardiovascular functional regulation, neurotransmission, inflammation, regulation of cell growth, apoptosis, and cardiovascular protection [5,6,7,8,9]. In addition, H_2_S scavenges various oxidants, such as superoxide and peroxynitrite, exhibits antioxidant effects against oxidative stress in multiple diseases [10,11]. In mammals, H_2_S is mainly generated with cysteine (Cys) or homocysteine (Hcy) degradation through the action of three main enzymes, cystathionine β-synthase (CBS), cystathionine γ-lyase (CSE) and 3-mercaptopyruvate sulphurtransferase (3-MST) [12]. Overexpression of H_2_S is related to various diseases like diabetes [13], tumors [14], liver cirrhosis [15,16], asthma [17], and myocardial damage [18]. That explains the increasing attention devoted to H_2_S detection and determination in living systems [16]. It is also worth mentioning that many of the processes in which hydrogen sulfide is involved remain unknown and should be further investigated to understand the precise role of H_2_S in pathological and psychological processes more clearly [19]. Therefore, developing promising and advanced methods for selective and sensitive detection of H_2_S is urgently needed.

Currently, two specific methods, chemical (iodometric, mercury, and methylene blue colorimetric) and physical processes (chromatography, laser, and H_2_S sensor), are available for detecting hydrogen sulfide [16]. These approaches are time-consuming, require complicated sample preparation, which often causes tissue or cell destruction [19], and most important are unsuitable for in situ analysis. It may seem that a good way to quantify H_2_S could be a fluorescence-based assay [20]. Various organic fluorescent probes have been explored recently due to advantages such as high sensitivity, specificity, minimal toxicities in cells and tissues, handy staining processes, and flexible molecular design strategies [21,22]. Fluorescent probes for H_2_S have been designed based on (i) azide-functionalized fluorophores reduction to amines [15,23,24,25,26], (ii) strong nucleophilic reaction [27,28], (iii) and the sulfide-induced precipitation [29,30,31]. In recent years, the development of fluorescence probes based on organic azide to detect H_2_S has been very intensive. Azide reduction is often rapid and produces large (10 ± 100-fold) fluorescence turn-ons, with the resultant probes exhibiting functional low micromolar detection limits and excellent selectivity profiles [32]. However, many defects still persist, including poor water solubility, unsatisfactory sensitivity, and potential photoreduction of azides to amines, which can lead to unwanted photoactivation in long-term imaging experiments. In addition, many probes are affected by the interference of small biological thiols (glutathione, cysteine, or homocysteine) because these biothiols show similar chemical properties as H_2_S [33].

The most common way to generate H_2_S for chemical and biological experiments is to use H_2_S donors [34]. Several series of donors with different release mechanisms have recently been developed [35]. The H_2_S-donating compounds compromise two major groups: (i) the inorganic salts such as NaHS or Na_2_S, which are rapid hydrogen sulfide releasers, and (ii) organic molecules associated with slow release of H_2_S like 4-methoxyphenyl)-morpholin-4-ylsulfanylidene-sulfido-λ5-phosphane;morpholin-4-ium (GYY4137) or diallyl trisulfide (DATS) [36].

In this research, we designed and synthesized an azide-based pro-fluorescent probe NAP-Py-N_3_ for rapid, highly selective, and sensitive detection of H_2_S with significant green fluorescence turn-on signal changes and large Stokes shift. NAP-Py-N_3_ displayed an emission peak at 380 nm. The presence of H_2_S could trigger a spontaneous reaction to form fluorescent NAP-Py-NH_2_ by reducing the azide group. Ultimately, the probe NAP-Py-N_3_ was applied to detect the dissociated and gaseous form of H_2_S.

## 2. Results and Discussion

### 2.1. Synthesis and Optical Characterizations of NAP-Py-N_3_

**Scheme 1:**
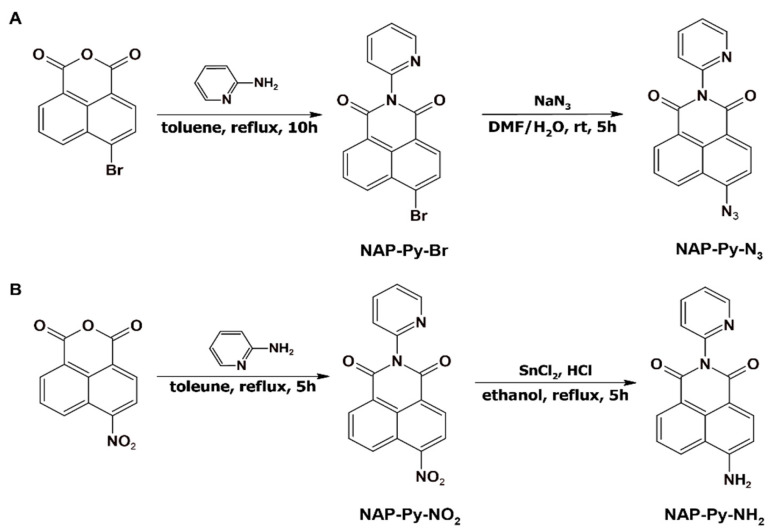
Synthesis route of (**A**) probe NAP-Py-N_3_ and (**B**) fluorescent standard NAP-Py-NH_2_.

In the probe design for H_2_S, 1,8-naphthalimide has been selected as an intramolecular charge transfer (ICT) fluorophore in light of its desirable spectroscopic properties, good stability, and feasibility in structure modification [37]. The azide group served as the H_2_S reduction reaction site. A novel naphthalimide pro-fluorescence material (NAP-Py-N_3_) with selectivity toward H_2_S was synthesized from commercially available 4-bromo-1,8-naphthalic anhydride in two steps, and the synthesis pathway is displayed in Scheme 1. Briefly, 4-bromo-1,8-naphthalic anhydride was first condensed with 2-aminopyridine to prepare NAP-Py-Br, which was subjected to a nucleophilic substitution reaction using NaN_3_ in DMF to produce the azido derivative NAP-Py-N_3_ as a yellow solid in 82% yield. NAP-Py-NH_2_ was obtained in two steps where 4-nitro-1,8-naphthalic anhydride was reacted with 2-aminopyridine to yield NAP-Py-NO_2_. The latter was subjected to a reduction via tin (II) chloride (SnCl_2_) to afford the desired amino product NAP-Py-NH_2_. All obtained compounds were thoroughly analyzed via ^1^H and ^13^C NMR and electrospray ionization mass spectrometry analyses (ESI-MS) (see Supplementary Materials, Figures S2–S13).

**Figure 1:**
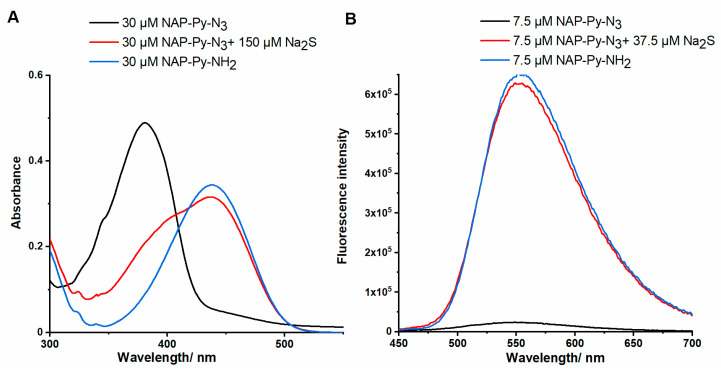
(**A**) UV-Vis absorption and (**B**) emission spectra of NAP-Py-N_3_, NAP-Py-NH_2_ after the bolus addition of Na_2_S to NAP-Py-N_3_ recorded in a phosphate buffer (0.1 M, pH 7.4) containing 10% of DMSO after 20 min incubation.

With the probe in hand, sensing properties toward H_2_S were first investigated through the measurement of UV-vis absorption and fluorescence emission spectra of NAP-Py-N_3_ in a phosphate buffer (PB buffer) solution (pH 7.4, 0.1 M, 10% DMSO) upon the addition of Na_2_S. As shown in Figure 1, the studied probe exhibited a maximum absorption wavelength at 380 nm, and a new absorption peak emerged at 435 nm upon the addition of Na_2_S, which can be attributed to the appearance of fluorophore NAP-Py-NH_2_. The influence of the azido group on photophysical properties is visible when we compare the fluorescence spectra of the probe and the corresponding sensing NAP-Py-NH_2_ product. NAP-Py-N_3_ is almost non-fluorescence, and after the reaction in the presence H_2_S, a strong fluorescence “turn-on” phenomenon is observed at 553 nm. Data presented in Table 1 show that the fluorescence quantum yield of the probe is much lower than the fluorescent standard. Notably, Na_2_S trigged a significant Stokes shift (118 nm). The results show that NAP-Py-N_3_ is a turn-on fluorescent probe for H_2_S in the PB buffer solution.

### 2.2. Time-Dependent Response of NAP-Py-N_3_ toward H_2_S

**Figure 2:**
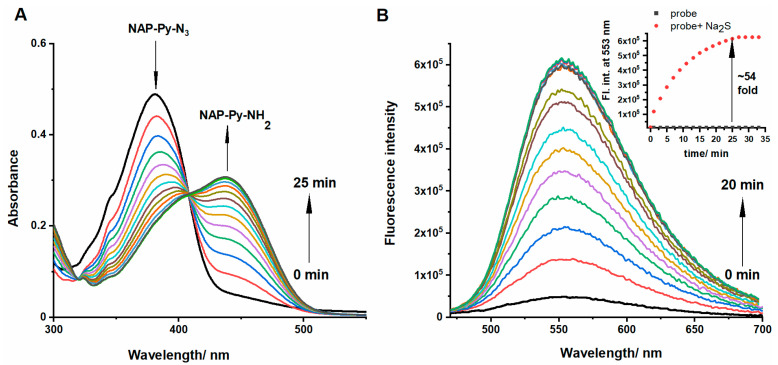
(**A**) Time-dependent absorbance spectra of NAP-Py-N_3_ (30 µM) recorded after addition of Na_2_S (150 µM). (**B**) Time-dependent fluorescence spectra (λ_ex_ 435 nm) of NAP-Py-N_3_ (7.5 µM) after addition of Na_2_S (37.5 µM). Inset: (**B**) Reaction profile of NAP-Py-N_3_ (7.5 µM) alone or in the presence of Na_2_S (37.5 µM) in PB buffer: DMSO (9:1, *v*/*v*).

Next, we assessed the spectral response of NAP-Py-N_3_ toward H_2_S by measuring the time-dependent absorption and fluorescence spectra of the probe solution upon the addition of five equivalents of Na_2_S (Figure 2). Recorded fluorescence and absorption spectra allow us to determine the time needed to release the maximum amounts of fluorescent NAP-Py-NH_2_ from the probe. A significant absorption peak is located at 435 nm and reaches the maximum within 25 min. After this time, no significant changes in absorption spectra were observed. Our probe reacted with the H_2_S source to yield a swift fluorescent response and after the first two minutes of the reaction, the fluorescence intensity increased several times, which may be sufficient to confirm the presence of hydrogen sulfide in various biosystems. The fluorescence peak at 553 nm reached a plateau within 20 min with a 54-fold enhancement. Moreover, no significant change in the probe’s fluorescence was observed without any analyte, indicating that NAP-Py-N_3_ is stable in an aqueous solution and may help detect H_2_S.

**Figure 3:**
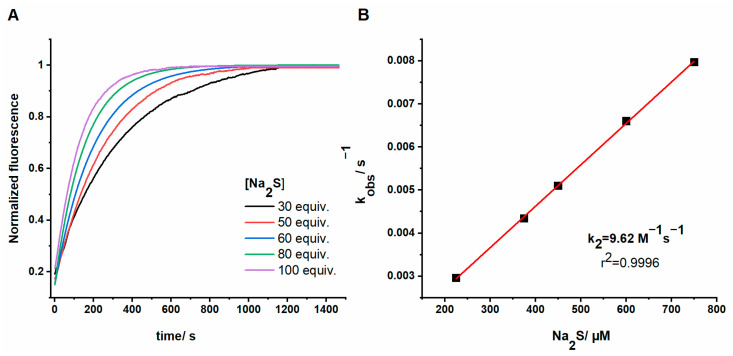
(**A**) The time-dependent fluorescence intensities at 553 nm for NAP-Py-N_3_ (7.5 µM) in the presence of different concentrations of Na_2_S. (**B**) The linear relationship of k_obs_ versus Na_2_S concentrations.

We determine the rate constant of the reaction of NAP-Py-N_3_ with H_2_S by measuring the fluorescent response of the NAP-Py-N_3_ probe after the addition of various amounts of Na_2_S (30–100 equivalents) in PB: DMSO solution (9:1, *v*/*v*). The time-dependent fluorescence at 553 nm was recorded for the analysis of data. The pseudo-first rate (k_obs_) was found after fitting the data with a single exponential function (Figure 3A). The reaction rate k_2_ (9.62 M^−1^s^−1^) was obtained via linear fitting of the k_obs_ versus Na_2_S concentration (Figure 3B).

### 2.3. Absorption and Fluorescence Titration

**Figure 4:**
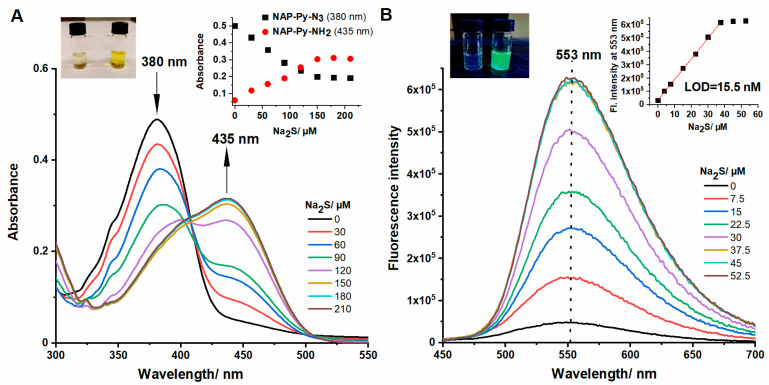
(**A**) Absorption spectral change in NAP-Py-N_3_ (30 µM) response to various concentrations of Na_2_S (0–210 µM) after 25 min incubation. (**B**) The fluorescence change in NAP-Py-N_3_ (7.5 µM) response to various concentration of Na_2_S (0–52.5 µM) after 20 min incubation. Inset: (**A**) The plot of absorbance changes at 380 and 435 upon the addition of Na_2_S; (**B**) fluorescence intensity changes at 553 nm of the probe as a function of Na_2_S concentrations.

Preliminary examinations revealed that NAP-Py-N_3_ could detect H_2_S via fluorescence and absorbance response. Sensitivity is an essential parameter for the fluorescence probe, so the absorption and fluorescence spectra of NAP-Py-N_3_ in the presence of different concentrations of Na_2_S in PB-DMSO (9:1 *v*/*v*, pH 7.4) were measured and is shown in Figure 4. The probe NAP-Py-N_3_ (30 µM) exhibited the maximum absorbance band at 380 nm (ε = 16,700 M^−1^cm^−1^). Upon the addition of various amounts of Na_2_S (0–210 μM), we observe three alternations: (i) the absorption band at 380 nm slowly decreases, (ii) simultaneously, the absorption band at 435 nm increases (ε = 11,500 M^−1^cm^−1^) and (iii) the solution color changes from faint yellow to deep yellow (Figure 4A). At the same time, an isoabsorption point at 403 nm was obtained. The five-fold and more significant excess of sodium sulfide used during titration do not affect the absorption spectrum of the probe, and even in the buffer solution, it was non-fluorescence. However, once encountered with increasing dosages of Na_2_S (from 0 to 7 equiv.), the probe exhibited a remarkable enhancement in emission in the green channel (553 nm) upon excitation at 435 nm (Figure 4B), corresponding to the generation of NAP-Py-NH_2_. In addition, the fluorescence intensity was linearly connected with the concentration of Na_2_S ranging from 0 to 37.5 µM for NAP-Py-N_3_. A linear equation (F_553 nm_ = a × [Na_2_S] μM +b, Figure 4B) was obtained between the concentration of Na_2_S and fluorescence intensity at 553 nm. Meanwhile, the R_2_ value of 0.9995 shows a consistent linear relationship among the variables. The detection limit of NAP-Py-N_3_ for H_2_S was calculated to be 15.5 nM according to the definition 3σ/k. This significant low detection limit implied that the probe was ultrasensitive toward Na_2_S, which further might be used to detect the intracellular and endogenous H_2_S, whose level is normally at low micromolar concentrations. In comparison to other fluorescence probes based on naphthalimides for H_2_S, we can conclude that our probe is much more sensitive than others (Table S1).

### 2.4. HPLC Titration

**Figure 5:**
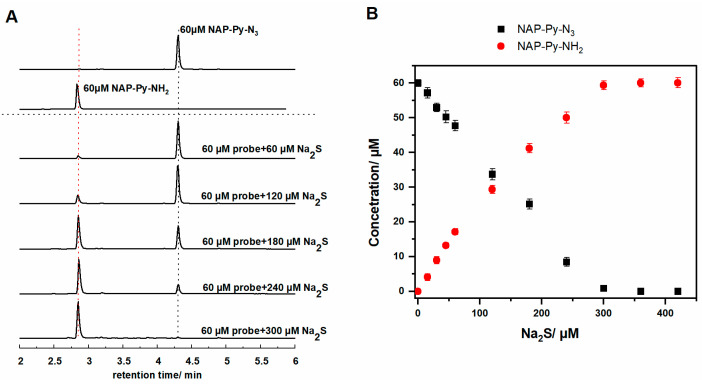
(**A**) HPLC chromatogram of standards and the reaction mixture of NAP-Py-N_3_ (60 µM) with Na_2_S (0–300 µM) after 25 min of incubation. (**B**) HPLC-based titration of probe (60 µM) with Na_2_S after 25 min of incubation in solution.

Our spectroscopic investigation showed that the fluorescent indicator is formed during the reaction of NAP-Py-N_3_ with Na_2_S. To detect and confirm that NAP-Py-NH_2_ is formed, we performed HPLC analyses. Moreover, HPLC titration of the probe with increasing concentration of Na_2_S was used to determine the reaction’s stoichiometry. HPLC chromatograms and stoichiometric analyses are depicted in Figure 5 and show the slow disappearance of the probe (NAP-Py-N_3_, r_t_ = 4.25 min). After adding the analyte to the solution of the probe, we observe the formation of a fluorescent product with a retention time of 2.8 min. A comparison of the retention time of the authentic standard of NAP-Py-NH_2_ confirms that this amino compound is the product found in the reaction mixture. Additionally, NAP-Py-NH_2_ is the sole product of the Na_2_S-induced probe conversion. Our analysis showed that five equivalents of Na_2_S completely consumed the NAP-Py-N_3_ probe.

### 2.5. Sensing Mechanism Studies

**Figure 6:**
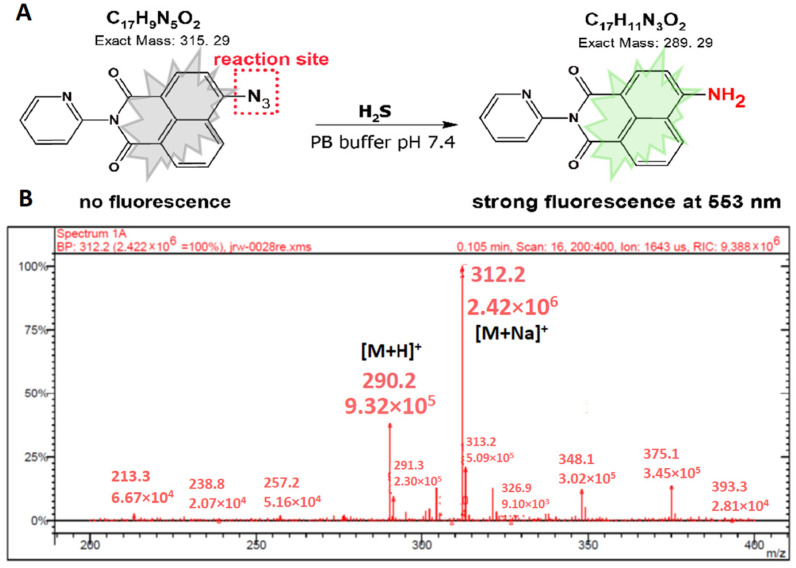
(**A**) The proposed detection mechanism of NAP-Py-N_3_ to H_2_S, (**B**) ESI-MS result of the isolated NAP-Py-NH_2_ from a reaction mixture of probe and excess of sodium sulfide. ESI-MS spectrum includes molecular ion peaks, the base peak, and the molecular ion isotopic peaks.

Subsequently, to obtain better insights into the proposed mechanism (Figure 6A), ESI-MS analysis was performed to confirm the sensing process of the probe NAP-Py-N_3_ with an excess of H_2_S. Based on our observation and knowledge, the NAP-Py-N_3_ probe possesses an azido group (-N_3_) as a single possible reaction site. The sulfide-mediated reduction in the aryl azide probe, after the initial nucleophilic attack of sulfide anion on the electrophilic azide, proceeds to form an azidethiol intermediate, followed by an intramolecular attack of sulfide to generate the proper fluorescence amine. Mass peaks at 290.2 and 312.2 *m*/*z* for [NAP-Py-NH_2_ + H]^+^ and [NAP-Py-NH_2_ + Na]^+^, respectively, was observed (Figure 6B), proving that the fluorophore NAP-Py-NH_2_ was indeed formed, which was consistent with our vision and could reasonably illustrate the sensing process of NAP-Py-N_3_ to H_2_S.

### 2.6. Selectivity and Co-Interference Studies of the Probe

**Figure 7:**
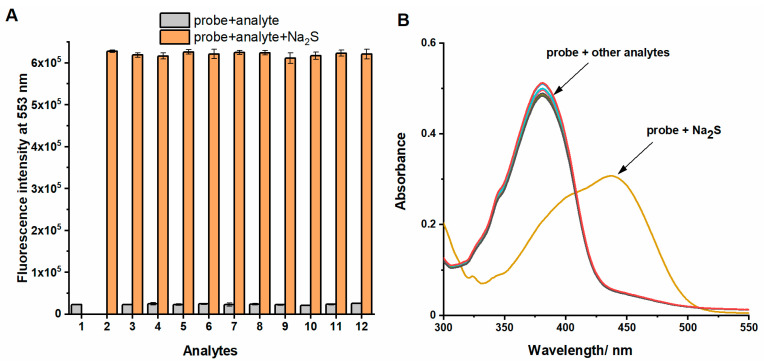
(**A**) Fluorescence spectra of NAP-Py-N_3_ (7.5 µM) toward Na_2_S and various species (37.5 µM for each). 1. Blank; 2. Na_2_S; 3. SO_4_^2−^; 4. SO_3_^2−^; 5. S_2_O_4_^2−^; 6. L-Cys; 7. HCy; 8. GSH; 9. NAC; 10. H_2_O_2_; 11. OCl^−^; 12. CN^−^. (**B**) Absorption spectra changes in NAP-Py-N_3_ (30 µM) with Na_2_S and various analytes (150 µM for each).

Based on the excellent stability of the probe and significant response, we subsequently carried out selectivity studies to confirm that the primary response is only observed for H_2_S. The selectivity of the probe was investigated through the fluorescent and absorption changes in the presence of various interfering species, including biothiol compounds (Cys, Hcy, GSH, and NAC) and different anions (SO_4_^2−^, SO_3_^2−^, S_2_O_4_^2−^, and CN^−^) (Figure 7). As expected, NAP-Py-N_3_ showed excellent selectivity toward H_2_S in the presence of various competing analytes. Only Na_2_S elicited a remarkable fluorescence enhancement at 553 nm and a new wide absorption band at 435 nm. Other interfering substances, including biothiols (L-Cys, GSH, Hcy, and NAC), hardly caused any noticeable fluorescence changes. This may be because the sensing reactions toward biothiols are much slower than Na_2_S. To check the interference of biothiols with coexistent H_2_S, we also tested NAP-Py-N_3_ with these analytes in the presence of Na_2_S. These findings suggested that all relevant analytes did not influence the detection of H_2_S, indicating the excellent anti-interference ability of NAP-Py-N_3_ for H_2_S recognition and its potential application in complex biological system imaging.

### 2.7. Detection of Gaseous H_2_S and Paper Test

**Figure 8:**
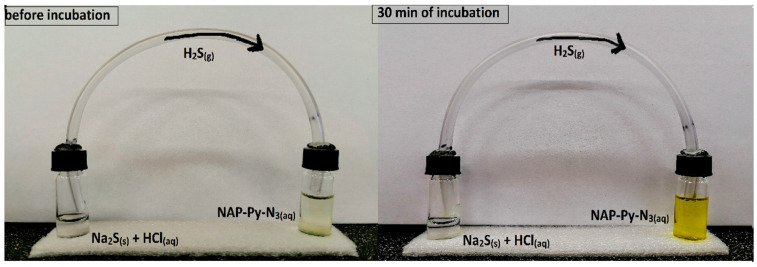
Colorimetric response of NAP-Py-N_3_ (100 µM) toward gaseous H_2_S before starting the experiment and after 30 min of incubation.

To improve the practicability of the probe, we checked whether the NAP-Py-N_3_ is sensitive toward the gaseous H_2_S. For this reason, we performed tests in solution and on the paper filter and we expected that the gaseous form of hydrogen sulfide would be able to reduce the azide group in the probe solution and release the fluorescent amine. In the experiment, we dissolved sodium sulfide in dilute hydrochloric acid in a glass vessel, and the vessel was immediately closed and connected with a plastic tube to a second glass vial containing a solution of 100 µM NAP-Py-N_3_ in PB (0.1 M pH 7.4, containing 10% DMSO). The color of the probe solution changed from a light yellow to a deep yellow (Figure 8) and the probe solution under UV light showed strong green fluorescence. These results indicate that gaseous H_2_S caused the reduction in the azide group. During this process, an amino-derived fluorophore was released. The probe can successfully detect H_2_S formation during the dissociation of, e.g., Na_2_S or NaHS, and can also confirm the presence of gaseous H_2_S released during various inorganic reactions or processes.

**Figure 9:**
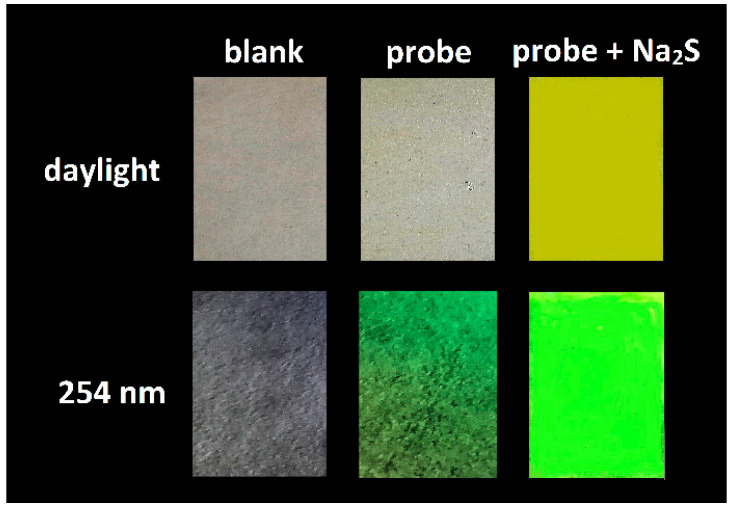
Photograph of filter paper color changes after treatment with H_2_S under daylight and light of 254 nm.

The test strips were prepared via immersing filter paper in PB-DMSO (*v*/*v* = 9/1) solution of NAP-Py-N_3_ (50 μM) and drying in air. Next, paper filters were placed in a sealed vessel with sodium sulfide. After that, dilute hydrochloric acid was added through a disposable syringe to prepare hydrogen sulfide gas. It was found that before and after exposure to saturated H_2_S vapor, the paper strips had a very significant color change, as shown in Figure 9. Under natural light conditions, we observed color changes, from colorless to yellow. Moreover, the paper strip, after exposure to UV light, showed tremendous green fluorescence. These results indicated that paper strips soaked with probe NAP-Py-N_3_ could detect gaseous H_2_S, and in the future, it could be used, for example, to monitor gas leak in chemical laboratories.

### 2.8. Monitoring the Level of H_2_S Generated from Organic Donors

**Figure 10:**
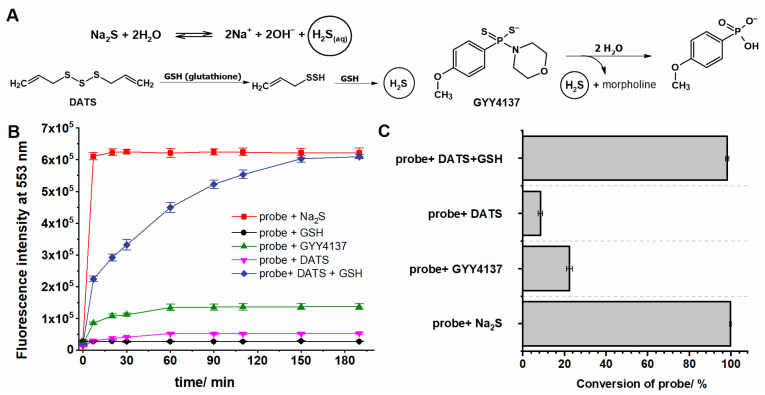
(**A**) Mechanism of H_2_S release from Na_2_S, GYY4137, and DATS. (**B**) The fluorescence changes at 553 nm during the reaction of the NAP-Py-N_3_ probe (7.5 µM) with H_2_S donors (750 µM for each). (**C**) Conversion of the NAP-Py-N_3_ probe after 180 min of reaction with the H_2_S donors.

Further research investigated whether our probe could detect H_2_S generated from recently reported organic hydrogen sulfide donors under physiological conditions. We have selected two representative examples of the donors based on different release mechanisms. The first one GYY4137, is a water-soluble compound that releases H_2_S via hydrolysis. According to the literature, this donor releases low levels of H_2_S at physiological pH. The second example is diallyl trisulfide (DATS), which rapidly releases hydrogen sulfide in a thiol-dependent reaction. Hydrolysis of DATS under aqueous conditions should not generate H_2_S [33,38]. The mechanism of hydrogen sulfide generation by organic donors is shown in Figure 10A.

Our studies have shown that the reaction of the probe NAP-Py-N_3_ with excess sodium sulfide caused an increase in fluorescence intensity, which reached its maximum after about 10 min. The addition of GYY4137 (10 equiv.) to the probe solution (PB: DMSO, 9:1, *v*/*v*, pH 7.4) in the first few minutes caused a slight increase in fluorescence, which remained at the same level throughout the experiment (i.e., for 3 h). Mixing the probe (7.5 µM) with DATS (750 µM), as expected, did not increase the fluorescence intensity at 553 nm even after 180 min. Only the addition of an activator such as glutathione (4-fold excess) to the mixture containing NAP-Py-N_3_ (7.5 µM) and DATS (750 µM) caused the inclusion of fluorescence, which increased with time and reached a plateau after 150 min (Figure 10B). Moreover, we do not observe any effect when we mixed the solution of the probe with an excess of glutathione.

Based on the obtained results, we can confirm that NAP-Py-N_3_ shows a rapid fluorescent response for H_2_S generated from Na_2_S, and the amount of H_2_S produced was sufficient for the complete reaction of the probe, which was confirmed in described studies using the HPLC technique. In stark contrast, GYY4137 generated a minimal amount of hydrogen sulfide under the same conditions, which allowed to reduce the amount of NAP-Py-N_3_ probe by about 20%. These results are consistent with other literature reports [36,39], which indicate a shallow level of H_2_S formation during the hydrolysis of GYY4137 at pH 7.4. Diallyl trisulfide reacts rapidly with glutathione to release H_2_S through the thiol-disulfide exchange. The addition of DATS (750 μM) to the GSH (4-fold excess) solution led to an instant production of H_2_S, which after 150 min allowed the probe to be wholly converted into a fluorescent NAP-Py-NH_2_ product (Figure 10C).

### 2.9. Influence of pH on the Fluorescence Response of NAP-Py-N_3_

To investigate whether the NAP-Py-N_3_ probe can be applied to monitor H_2_S in biological systems, the probe with Na_2_S was tested under different pH conditions. To study the effect of pH, the fluorescence intensity of the probe (7.5 µM) at 553 nm before and after adding Na_2_S (37.5 µM) was explored in the pH range from 3.5 to 10.5 (Figure S1). The probe NAP-Py-N_3_ is very stable over the given pH range, and significant fluorescence enhancement was detected at 553 nm with the addition of Na_2_S under the pH range from 7 to 10.5. However, in strongly acidic conditions (pH < 5), the fluorescence intensity of the mixture is the lowest. This phenomenon may be explained with three reasons. The reduction in the -N_3_ group does not occur since there is almost no HS^−^ in the robust acid solution. The higher H^+^ ion concentration will decrease the reaction speed between the probe and HS^−^. In addition, the higher H^+^ ion concentration will directly decrease the fluorescence intensity of NAP-Py-NH_2,_ which is the reaction product of the probe and H_2_S. The solution of NAP-Py-NH_2_ should also exhibit a downward trend with the decrease in pH in the range of 3.5–6.5. At a lower pH, the amino group of fluorescence standard will be protonated, leading to the decrease in fluorescence intensity at 553 nm. The alkaline solution increases the ionization of H_2_S and restores the fluorophore. The results indicate that NAP-Py-N_3_ can detect H_2_S under physiological conditions and may be used to monitor hydrogen sulfide formation in the biological system.

## 3. Materials and Methods

### 3.1. Materials and Instruments

All chemical reagents were purchased from commercial suppliers and used without further purification. Distilled water was used throughout all experiments. Column chromatography was performed on silica gel 60 (70–230 mesh ASTM). The progress of the reactions was monitored with TLC silica plates (60-F_254_). ^1^H and ^13^C NMR spectra were recorded on a Bruker Avance III 600 spectrometer using DMSO-*d_6_*. The chemical shifts (*δ*) are reported in ppm and coupling constants (J) are reported in Hz. Mass spectrometric data were obtained from Varian 500-MS LC Ion Trap. UV-vis absorption data were recorded on a Jasco V-670 spectrophotometer, and fluorescence spectra were determined using a spectrofluorometer FLS920 (Edinburgh Instruments, UK). HPLC chromatograms were collected using UFLC Shimadzu equipped with a UV-vis absorption and fluorescence detector. Analysis was performed using a Kinetex C18 column (Phenomenex 100 mm × 46 mm, 2.6 µm) equilibrated with 10% of acetonitrile (MeCN) in water containing 0.1% trifluoroacetic acid (TFA). The quantum yields of the probe NAP-Py-N_3_ and fluorescence standard NAP-Py-NH_2_ were determined using fluorescein as a reference (ϕ = 0.79 [40] in 0.1 M NaOH). To evaluate hydrogen sulfide concentration in the phosphate buffer, we used Ellman’s reagent, i.e., 5,5′-dithiobis (2-nitrobenzoic acid) (DTNB); the detailed procedure is described in our previous paper [41]. 

### 3.2. Synthesis of Probe NAP-Py-N_3_

#### 3.2.1. Synthesis of N-Pyridinyl-4-Bromo-1,8-Naphthalimide (NAP-Py-Br) [42]

4-Bromo-1,8-naphthalic anhydride (1.656 g, 6 mmol) was dissolved in toluene (45 mL) and then 2-aminopyridine (1.694 g, 18 mmol) was added into the solution. The reaction mixture was refluxed for 10 h. After completion of the reaction, the mixture was cooled down to room temperature, and the precipitate was collected via filtration under reduced pressure. After drying, the crude product was recrystallized from ethanol to yield a pure compound, a brown powder (1.63 g, 77%). ^1^H NMR (DMSO-*d_6_*, 600 MHz) *δ* (ppm): 7.56–7.61 (m, 2H); 8.05–8.08 (m, 2H); 8.28 (d, 1H, J = 7.8 Hz); 8.37 (d, 1H, J = 7.8 Hz); 8.61–8.67 (m, 3H); ^13^C NMR (DMSO-*d*_6_, 151 MHz) *δ* (ppm): 122.68; 123.47; 124.79; 124.87; 129.38; 129.44; 130.15; 130.57; 131.61; 132.02; 132.25; 133.63; 139.24; 149.88; 149.91; 163.45; 163.52; ESI-MS *m/z* (M + H)^+^ 355.1 for C_17_H_9_BrN_2_O_2_.

#### 3.2.2. Synthesis of NAP-Py-N_3_

NAP-Py-Br (1.0 g, 2.8 mmol) was dissolved in 20 mL DMF, and NaN_3_ (1.82 g, 30 mmol) was added in one portion. The prepared mixture was stirred for 4 h at 40 °C in the dark. Next, the reaction mixture was poured into 50 mL of ice water, and then the resulting precipitate was filtered off and washed with cold water (20 mL). After drying, the residue was purified with silica gel chromatography using CH_2_Cl_2_: MeOH (17:3) as the eluent to afford a pure compound, a yellow solid (0.72 g, 82%). ^1^H NMR (DMSO-*d_6_*, 600 MHz) *δ* (ppm): 7.55–7.59 (m, 2H); 7.81 (d, 1H, J = 7.8 Hz); 7.90–7.93 (m, 1H); 8.04–8.07 (m, 1H); 8.49–8.52 (m, 2H); 8.55–8.56 (m, 1H); 8.65–8.66 (m, 1H); ^13^C NMR (DMSO-*d_6_*, 151 MHz) *δ* (ppm): 116.60; 118.71; 122.81; 124.26; 124.69; 124.92; 127.89; 129.36; 129.37; 132.15; 132.24; 139.18; 143.89; 149.89; 150.09; 163.36; 163.84; ESI-MS *m/z* (M + H)^+^ 316.2 for C_17_H_9_N_5_O_2_.

### 3.3. Synthesis of Fluorescent Amino Derivative Standard NAP-Py-NH_2_

#### 3.3.1. Synthesis of NAP-Py-NO_2_

4-Nitro-1,8-naphthalic anhydride (0.24 g, 1.0 mmol) and 2-aminopyridine (0.38g, 4 mmol) were dissolved in anhydrous toluene and the mixture was refluxed for 5 h. The resulting precipitate was filtered off, washed with toluene, and dried under a vacuum. The dry crude product was recrystallized from ethanol to yield a pure compound, an orange powder (0.26 g, 81.5%). ^1^H NMR (DMSO-*d_6_*, 600 MHz) *δ* (ppm): 7.58–7.61 (m, 2H); 8.07–8.15 (m, 2H); 8.58–8.68 (m, 4H); 8.37 (d, 1H, J = 7.8 Hz); 8.61–8.67 (m, 1H); ^13^C NMR (DMSO-*d_6_*, 151 MHz) δ (ppm): 123.34; 123.41; 124.75; 124.79; 124.94; 127.19; 129.33; 129.68; 130.31; 130.65; 132.32; 139.33; 149.59; 149.95; 149.97; 162.67; 163.48; ESI-MS *m/z* (M + H)^+^ 320.2 for C_17_H_9_N_3_O_4_.

#### 3.3.2. Synthesis of NAP-Py-NH_2_

NAP-Py-NO_2_ (0.11 g, 0.35 mmol), 30 mL of absolute ethanol, stannous chloride (0.329 g, 1.75 mmol) and 1 mL of concentrated hydrochloric acid (35%) were added to a 50 mL flask, and the magnetically stirred mixture was heated under reflux. The progress of the reaction was monitored via TLC (CH_2_Cl_2_: MeOH, 17:3). After completion of the reaction (5 h), the mixture was concentrated to approx. 15 mL, and the resulting precipitate was collected via filtration, washed with 1% aqueous NaOH (2 mL × 2) and dried under vacuum. The residue was purified using silica gel chromatography with CH_2_Cl_2_: MeOH (17:3) as the eluent to yield an orange solid (80 mg, 79%). ^1^H NMR (DMSO-*d_6_*, 600 MHz) *δ* (ppm): 6.90 (d, 1H, J = 7.8 Hz); 7.49–7.54 (m, 4H); 7.70 (t, 1H, J = 7.8 Hz); 7.99–8.02 (m, 1H); 8.20 (d, 1H, J = 7.8 Hz); 8.44 (d, 1H, J = 7.8 Hz); 8.63 (d, 1H, J = 4.2 Hz); 8.69 (d, 1H, J = 8.4 Hz); ^13^C NMR (DMSO-*d_6_*, 151 MHz) *δ* (ppm): 107.93; 108.80; 120.06; 122.46; 124.26; 124.22; 125.04; 130.27; 130.87; 131.62; 134.47; 138.89; 149.74; 150.88; 153.61; 163.48; 164.41; ESI-MS *m/z* (M + H)^+^ 290.2 for C_17_H_11_N_3_O_2_.

### 3.4. General Procedure for Spectroscopic and HPLC Studies

All spectra were measured in a phosphate buffer (PB)-dimethyl sulfoxide (DMSO) (0.1 M, pH 7.4, *v*/*v* = 9:1) solution. Probe NAP-Py-N_3_ and fluorescence standard NAP-Py-NH_2_ were dissolved in DMSO to prepare an 1 mM stock solution. Before measurements, samples were diluted with PB to afford the final concentrations. Sodium sulfide (Na_2_S) was used as an H_2_S source and the 1 mM stock solution in PB buffer was always prepared before each experiment. To assess the selectivity of the probe, aqueous stock solutions (1 mM) of sodium salts (SO_4_^2−^, SO_3_^2−^, S_2_O_4_^2−^, and CN^−^), amino acids (L-Cys, HCy, GSH, and NAC) and oxidants (H_2_O_2_ and OCl^−^) were used. The stock solutions of GYY4137 and DATS (10 mM) were prepared in DMSO. All experiments were performed in a 3.5 mL quartz cuvette with a 2 mL solution. For the measurements of fluorescence spectra, unless otherwise stated, the excitation was performed at 435 nm, and the slit widths for excitation and emission are set to 1.5 nm. Three parallel samples were prepared for each specific experimental condition, and the spectra were recorded three times. The HPLC traces of NAP-Py-N_3_ and NAP-Py-NH_2_ formed in the probe reaction with Na2S were detected by monitoring absorption at 380 nm. The samples were eluted by increasing the concentration of MeCN from 10 to 100% over 12 min at a flow rate of 1.5 mL per minute.

## 4. Conclusions

A novel probe NAP-Py-N_3_ based on a naphthalimide core has been designed and synthesized. The probe can rapidly respond to H_2_S in an aqueous solution with noticeable colorimetric and fluorescent changes. The interaction of NAP-Py-N_3_ with H_2_S reduced the azide group to amine and generated a concealed emission of NAP-Py-NH_2_. This emerging bright green emission was also accompanied by a 110 nm Stokes shift. The H_2_S-instigated azide reduction was evidenced with different spectroscopic studies, and photo-physical outcomes were further proved using HPLC chromatograms as well as NMR or MS spectra. The probe showed excellent selectivity toward H_2_S with high sensitivity (LOD = 15.5 nM). We believe that the NAP-Py-N_3_ probe could be convenient and efficient for monitoring H_2_S levels in various environmental and biological samples.

## Figures and Tables

**Table 1:** Spectroscopic properties of NAP-Py-N_3_ and NAP-Py-NH_2_ in PB: DMSO (0.1 M, pH 7.4, 9:1, *v*/*v*).

Compound	λ_max_ (nm)	ε (M^−1^cm^−1^)	λ_exc_ (nm)	λ_em_ (nm)	ϕ_em_ (%)	Stokes Shift (nm)
NAP-Py-N_3_	380	16,700	435	553	0.32	173
NAP-Py-NH_2_	435	11,500	435	553	36.1	118

## Data Availability

Not applicable.

## Supplementary Materials

The following are available online at https://www.mdpi.com/article/10.3390/molecules28176299/s1. The document provides additional data, including ^1^H, ^13^C NMR, ESI-MS spectra, and spectrophotometric study [25,43,44,45,46,47,48,49,50] (Table S1, Figures S1–S13).

## Sample Availability

Samples of all the synthesized compounds are not available from the authors.
