# Supplementary material for: Sensitive Detection of Various Forms of Hydrogen Sulfide via Highly Selective Naphthalimide-Based Fluorescent Probe
Source: Molecules. 2023 Aug 28;28(17):6299. doi: 10.3390/molecules28176299 (PMC10488666; doi:10.3390/molecules28176299)
Supplement: Supplementary file 1 [file molecules-28-06299-s001.zip › molecules-2554021-supplementary.pdf]

Supplementary Materials
Sensitive Detection of Various Forms of Hydrogen Sulfide by Highly Selective Naphthalimide-Based Fluorescent Probe

Daniel Słowiński [1], Małgorzata Świerczyńska [1], Jarosław Romański [2] and Radosław Podsiadły [1,*]

[1] Institute of Polymer and Dye Technology, Faculty of Chemistry, Lodz University of Technology, Stefanowskiego 16, 90-537 Lodz, Poland; daniel.slowinski@dokt.p.lodz.pl (D.S.); malgorzata.swierczynska@dokt.p.lodz.pl (M.Ś.)
[2] Department of Organic and Applied Chemistry, Faculty of Chemistry, University of Lodz, Tamka 12, 91-403 Lodz, Poland; jaroslaw.romanski@chemia.uni.lodz.pl
* Correspondence: radoslaw.podsiadly@p.lodz.pl

Table S1. Comparison of **NAP-Py-N3** with the fluorescent probes previously reported.

Structures	Medium	ϕ_{probe}	λ_{em} (nm)	λ_{ex} (nm)	k_2 ($M^{-1}s^{-1}$)	LOD (nM)	Ref.
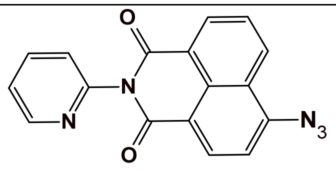 **NAP-Py-N3**	PB:DMSO (9:1, *v*/*v*)	0.003	553	435	9.62	15.5	Our work
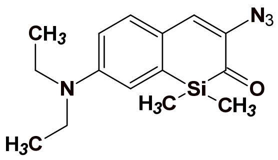 **SicAz2**	PBS:MeCN (7:3, *v*/*v*)	0.002	515	404	ND*	300	[S1]
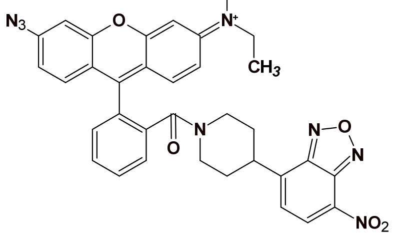	PBS buffer (20 mM, pH 7.4)	0.0250	565	530	1.60	120	[S2]

**probe 1**							
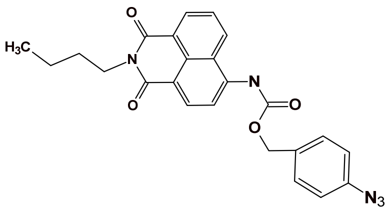 **NAP-1**	PBS:MeCN (19:1, *v*/*v*)	0.11	541	415	5.0	110	[S3]
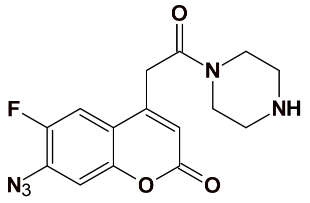 **7b**	PBS:DMF (9:1, *v*/*v*)	0.012	450	350	4.16	610	[S4]
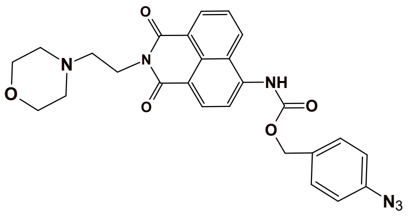 **LR-H_2S**	PBS:MeCN (4:1, *v*/*v*)	0.12	541	410	ND*	700	[S5]
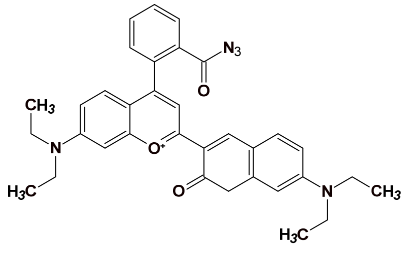 **1**	H_2O:acetone (7:3, *v*/*v*)	0.56	478	410	ND*	220	[S6]
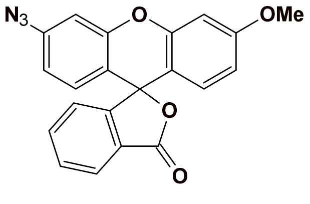	50 mM PIPES buffer, 100 mM KCl, pH 7.4	<0.01	516	476	ND*	86	[S7]

**MeRho-Az**							
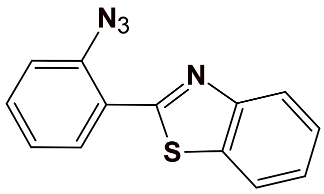 **1**	PBS buffer (10 mM, pH 7.4, 1 mM CTAB)	0.0064	450	375	ND*	0.78	[S8]
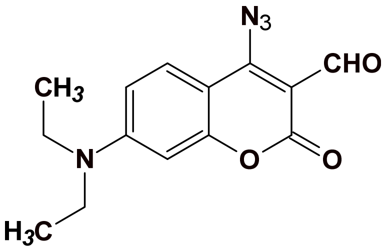 **CA**	MeCN:H_2O (3:7, *v*/*v*)	422		360	ND*	3.5	[S9}

*ND-no determined

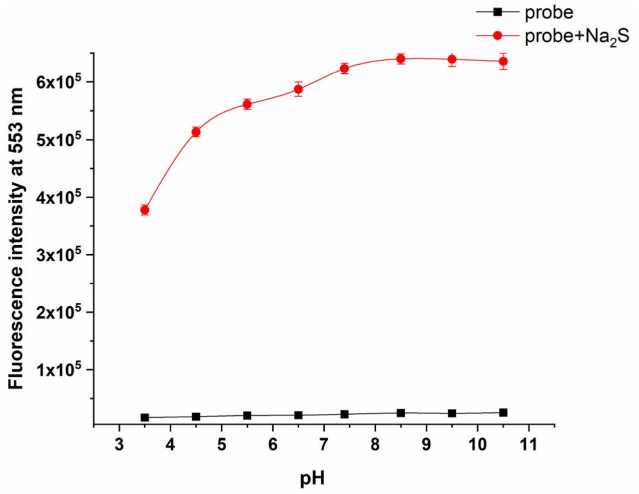

Figure S1. The fluorescence intensity of **NAP-Py-N$_3$** (7.5 μM) in the absence and presence of Na_2S (37.5μM) in different pH buffer solutions (excitation is 435 nm, slites 1.5/1.5 nm). Data are shown as mean ± standard deviation of three independent experiments.

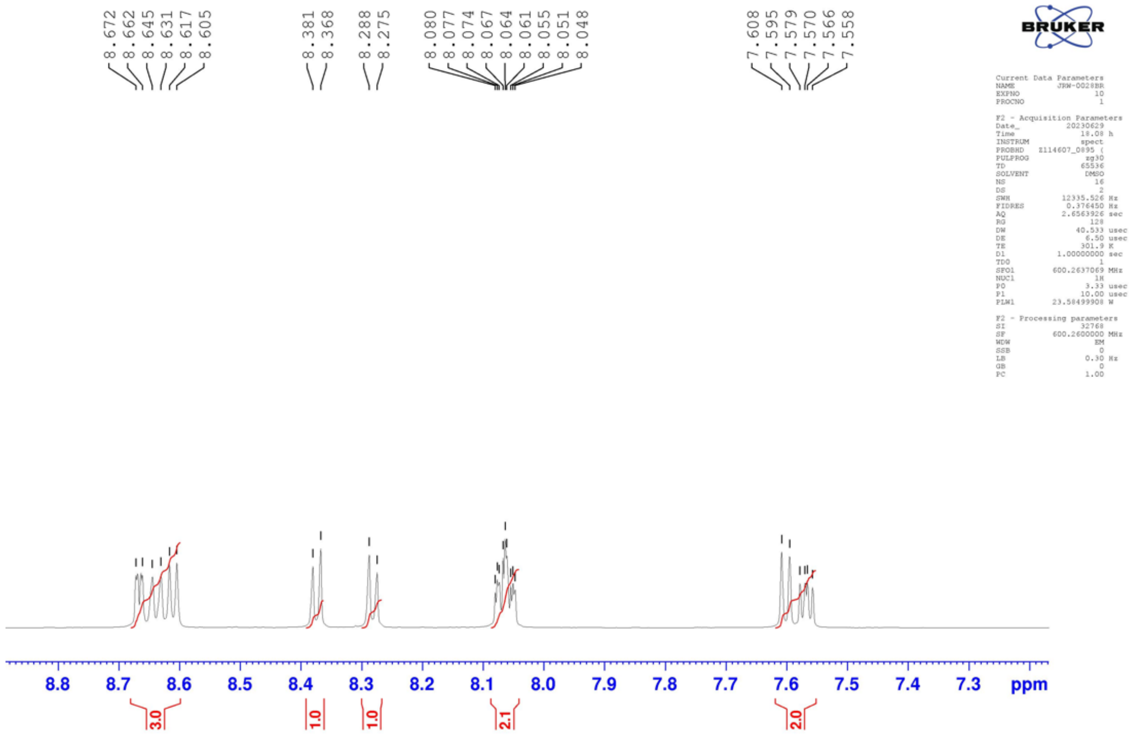

Figure S2. ^{1}H-NMR spectra of **NAP-Py-Br**

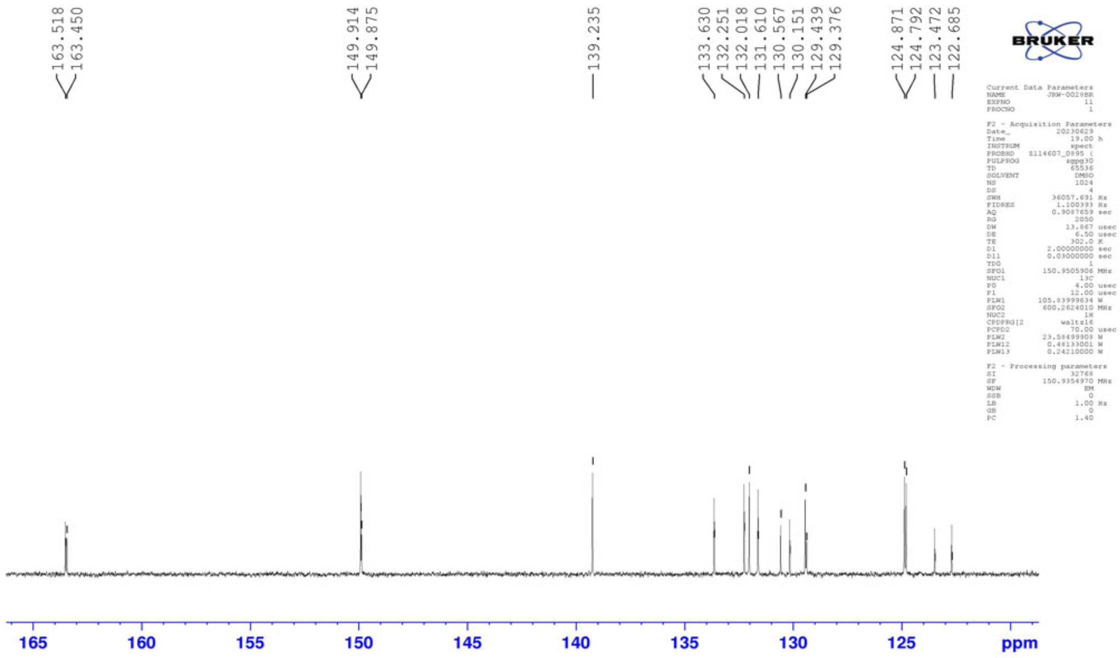

Figure S3. ^{13}C-NMR spectra of **NAP-Py-Br**

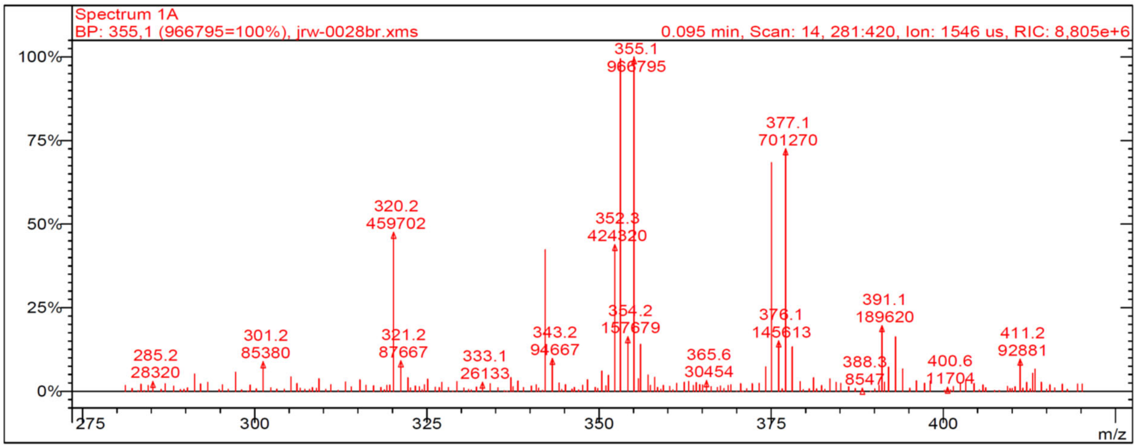

Figure S4. ESI-MS spectra of **NAP-Py-Br**

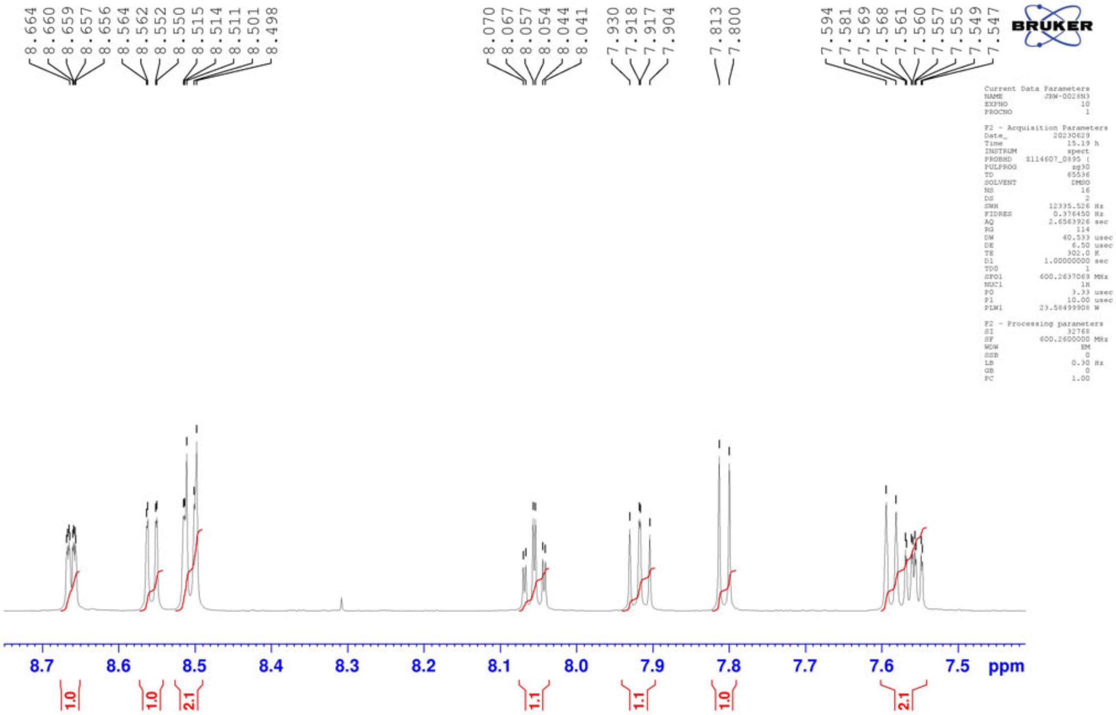

Figure S5. ^{1}H-NMR spectra of **NAP-Py-N$_3$**

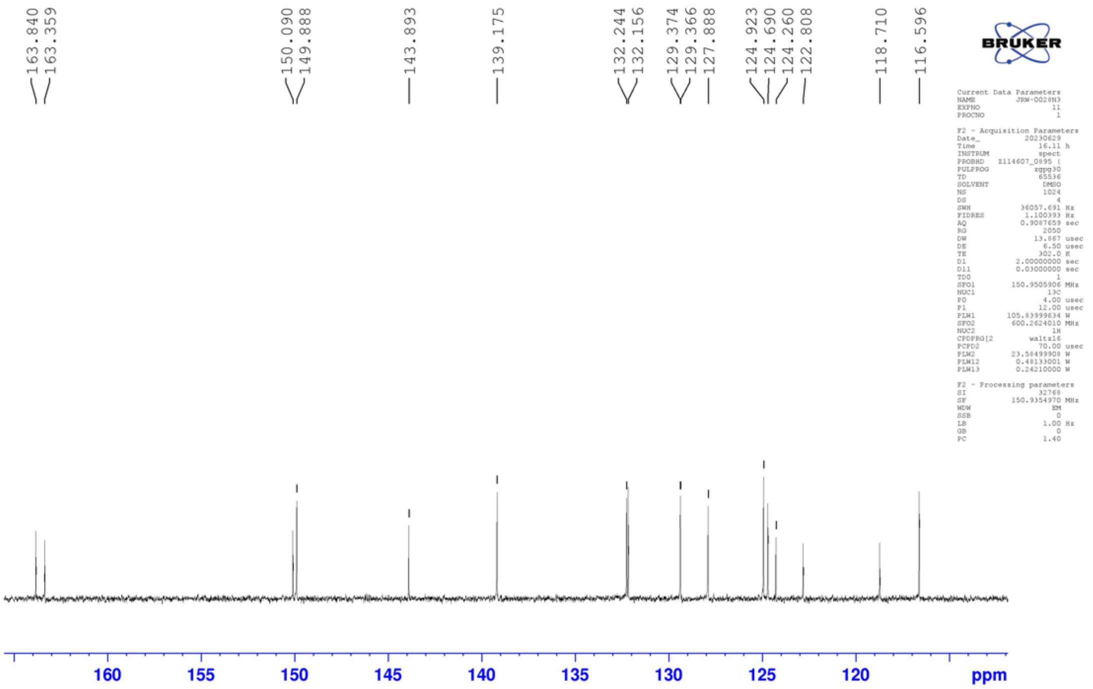

Figure S6. ^{13}C-NMR spectra of **NAP-Py-N$_3$**

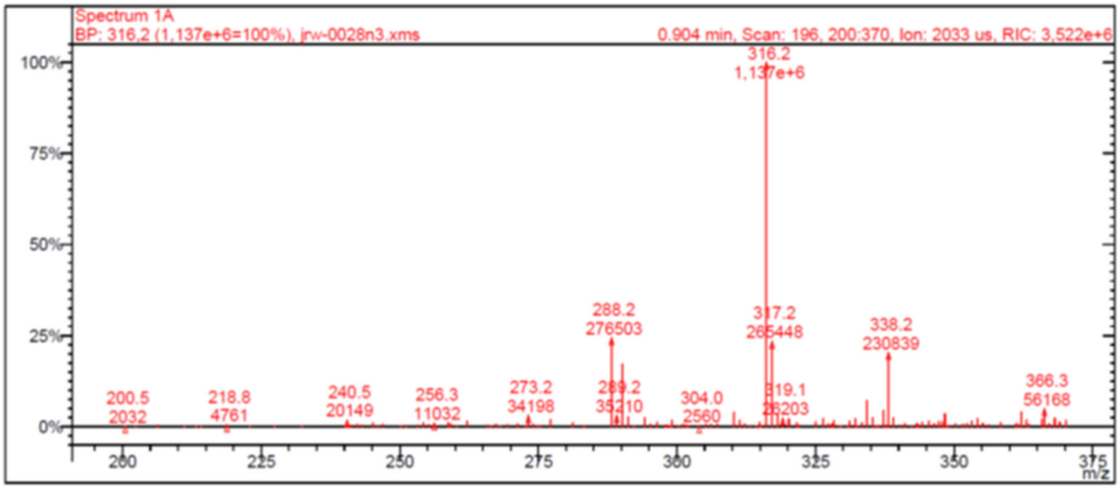

Figure S7. ESI-MS spectra of **NAP-Py-N$_3$**

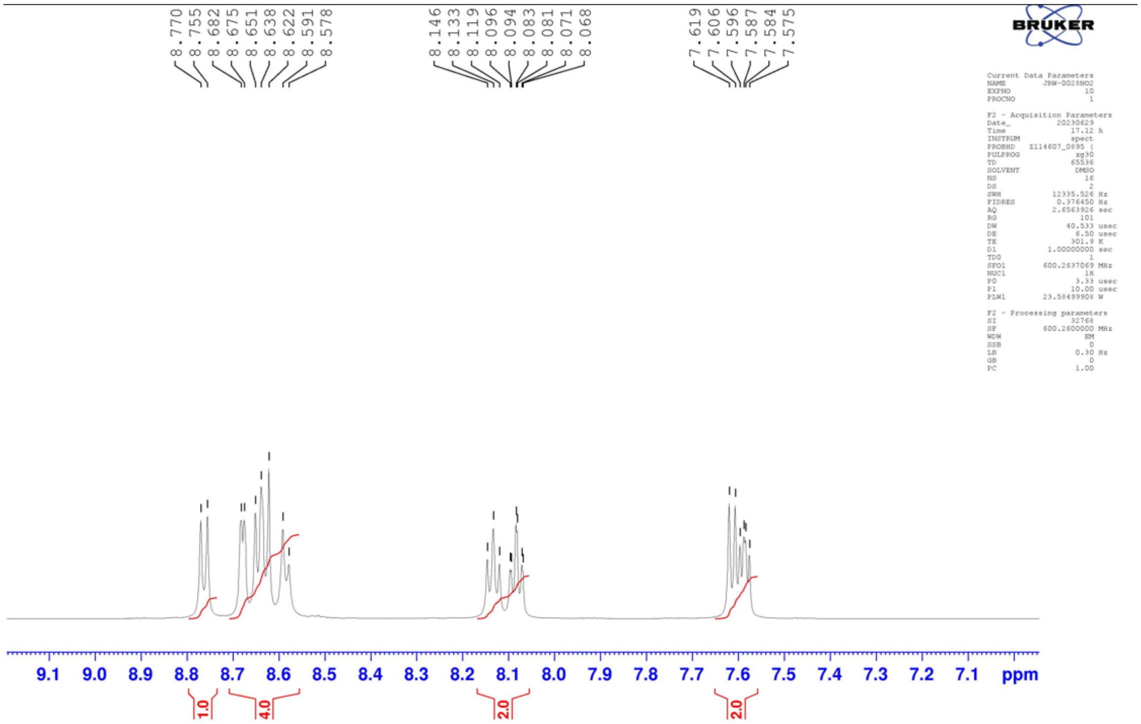

Figure S8. ^{1}H-NMR spectra of **NAP-Py-NO$_2$**

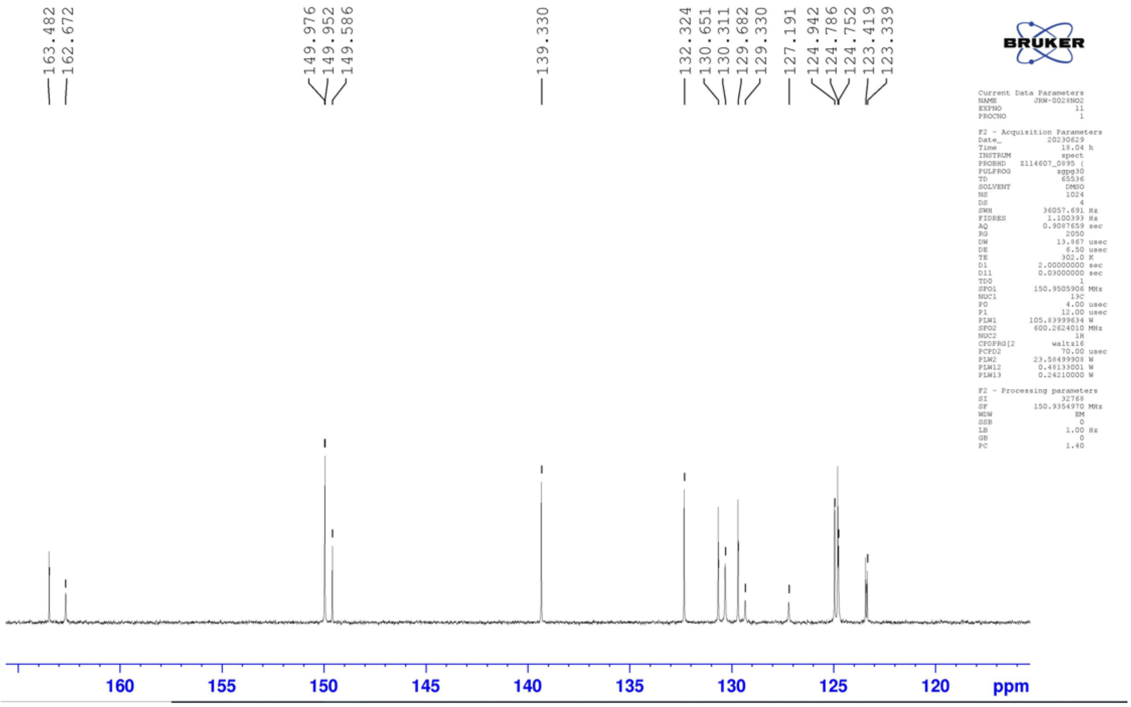

Figure S9. ^{13}C-NMR spectra of **NAP-Py-NO$_2$**

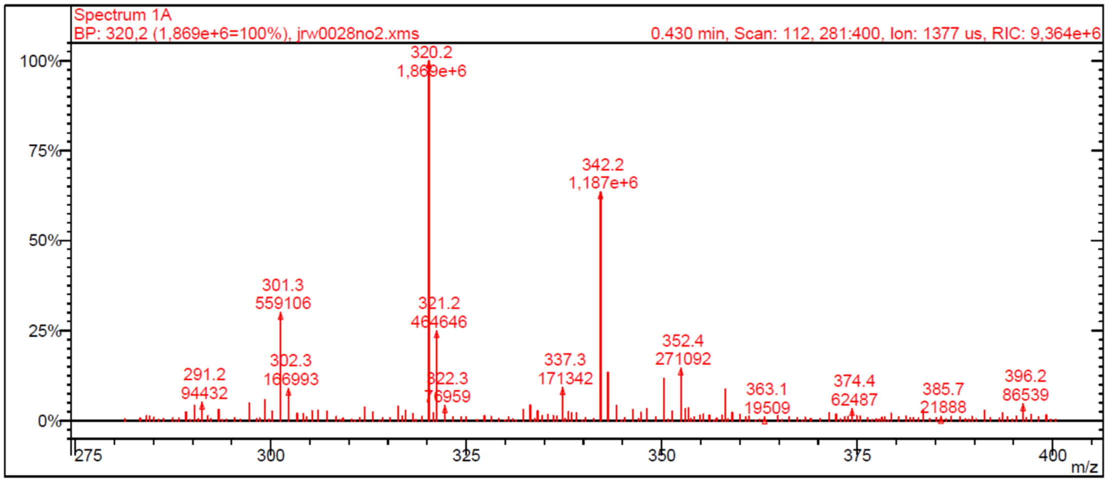

Figure S10. ESI-MS spectra of **NAP-Py-NO₂**

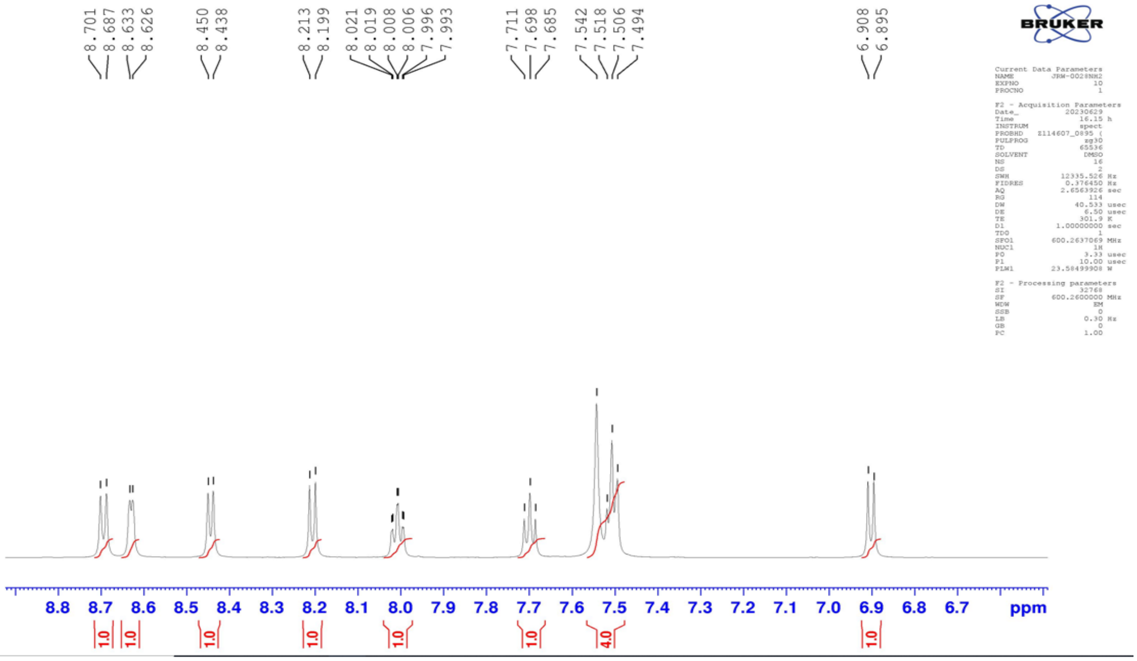

Figure S11. ^{1}H-NMR spectra of **NAP-Py-NH₂**

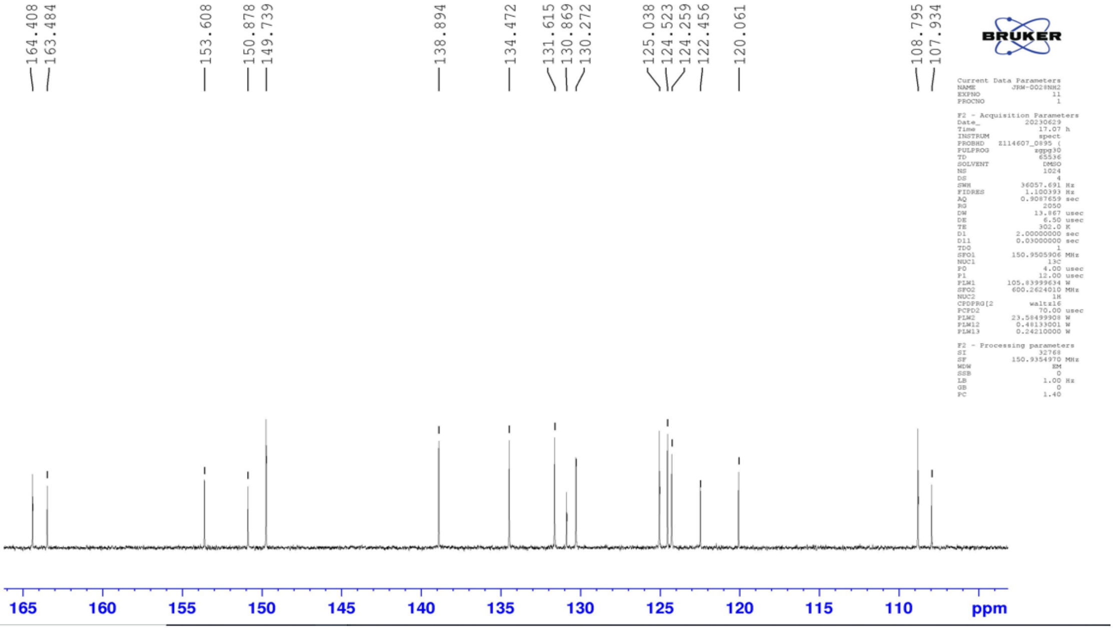

Figure S12. ^{13}C-NMR spectra of **NAP-Py-NH$_2$**

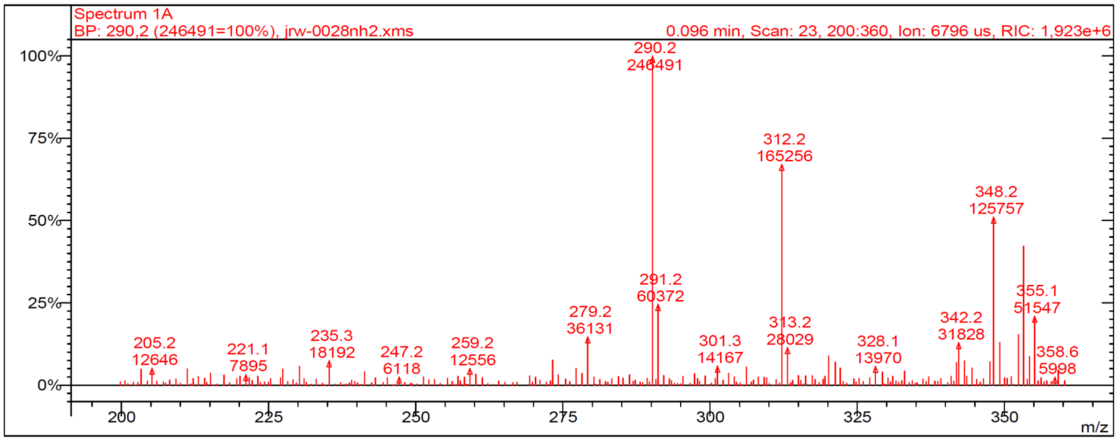

Figure S13. ESI-MS spectra of **NAP-Py-NH$_2$**

S1. Sun, Y., Li, C., Feng, X., Wang, C., Wang, N., Zhu, J., Wang, T., Cui, X. Si-coumarin-based fluorescent probes for ultrafast monitoring H_2S in vivo. *Dyes and Pigments* **2021**, *186*, 109059.

S2. Ma, C.; Wei, C.; Li, X; Zheng, X,; Chen, B.; Wang, M.; Zhang, P.; Li, X. A mitochondria-targeted dual-reactable fluorescent probe for fast detection of H_2S in living cells. *Dyes and Pigments* **2019**, *162*, 624-631.

S3. Zhang, L.; Li, S.; Hong, M.; Xu, Y.; Wang, S.; Liu, Y.; Qian, Y.; Zhao, J. A colorimetric and ratiometric fluorescent probe for the imaging of endogenous hydrogen sulphide in living cells and sulphide determination in mouse hippocampus. *Org. Biomol. Chem.* **2014**, *12*, 5115.

S4. Wei, C.; Wang, R.; Wei, L.; Cheng, L.; Li, Z.; Xi, Z.; Yi, L. O-fluorination of aromatic azides yields improved azido-based fluorescent probes for hydrogen sulfide: synthesis, spectra, and bioimaging. *Chem. Asian J.* **2014**, *9*, 3586 – 3592.

S5. Feng, W.; Mao, Z.; Liu, L.; Liu, Z. A ratiometric two-photon fluorescent probe for imaging hydrogen sulfide in Lysosomes. *Talanta* **2017**, *167*, 134-142.

S6. Duan, Y.; Yang, X.; Zhong, Y.; Guo, Y.; Li, Z.; Li, H. A ratiometric fluorescent probe for gasotransmitter hydrogen sulfide based on a coumarin-benzopyrylium platform. *Anal. Chim. Acta* **2015**, *859*, 59-65.

S7. Hammers, M.; Taormina, M.; Cerda, M.; Montoya, L.; Seidenkranz, D.; Parthasarathy, R.; Pluth, M. Bright. fluorescent probe for H_2S enables analyte-responsive, 3D imaging in live zebrafishu light sheet fluorescence microscopy. *J. Am. Chem. Soc.* **2015**, *137*, 10216−10223.

S8. Zhang, J.; Guo, W. A new fluorescent probe for gasotransmitter H_2S: high sensitivity, excellent selectivity, and a significant fluorescence off–on response. *Chem. Commun.* **2014**, *50*, 4214-4217.

S9. Fang, Q.; Xiong, H.; Yang, L.; Wang, B.; Song, X. An instantaneous fluorescent probe for detecting hydrogen sulfide in biological systems. *New J. Chem.* **2019**, 43, 13594-13599.
